# Inefficient skeletal muscle oxidative function flanks impaired motor neuron recruitment in Amyotrophic Lateral Sclerosis during exercise

**DOI:** 10.1038/s41598-017-02811-z

**Published:** 2017-06-07

**Authors:** F. Lanfranconi, A. Ferri, G. Corna, R. Bonazzi, C. Lunetta, V. Silani, N. Riva, A. Rigamonti, A. Maggiani, C. Ferrarese, L. Tremolizzo

**Affiliations:** 1 School of Medicine and Surgery and Milan Center for Neuroscience (NeuroMI), University of Milano-Bicocca, Milano, Italy; 2 Clinical Exercise Science Research Program, Institute of Sport, Exercise and Active Living (ISEAL), Victoria University, Melbourne, Australia; 3 NEuroMuscular Omnicentre (NEMO), Fondazione Serena Onlus, Milano, Italy; 4 Department of Neurology and Laboratory Neuroscience – IRCCS Istituto Auxologico Italiano, Pioltello, Italy; 5 Department of Pathophysiology and Transplantation, Dino Ferrari Centre, Università of Milan, Milano, Italy; 6 San Raffaele Hospital, Milano, Italy; 7 Alessandro Manzoni Hospital, Lecco, Italy; 8 Italian Academy of Osteopathic Medicine (AIMO), Saronno, Italy; 9 Neurology Unit, “San Gerardo” Hospital, Monza, Italy

## Abstract

This study aimed to evaluate muscle oxidative function during exercise in amyotrophic lateral sclerosis patients (pALS) with non-invasive methods in order to assess if determinants of reduced exercise tolerance might match ALS clinical heterogeneity. 17 pALS, who were followed for 4 months, were compared with 13 healthy controls (CTRL). Exercise tolerance was assessed by an incremental exercise test on cycle ergometer measuring peak O_2_ uptake ($\dot{{\rm{V}}}$O_2peak_), vastus lateralis oxidative function by near infrared spectroscopy (NIRS) and breathing pattern ($\dot{{\rm{V}}}$E _peak_). pALS displayed: (1) 44% lower $\dot{{\rm{V}}}$O_2peak_ *vs*. CTRL (p < 0.0001), paralleled by a 43% decreased peak skeletal muscle oxidative function (p < 0.01), with a linear regression between these two variables (r^2^ = 0.64, p < 0.0001); (2) 46% reduced $\dot{{\rm{V}}}$E_peak_ *vs*. CTRL (p < 0.0001), achieved by using an inefficient breathing pattern (increasing respiratory frequency) from the onset until the end of exercise. Inefficient skeletal muscle O_2_ function, when flanking the impaired motor units recruitment, is a major determinant of pALS clinical heterogeneity and working capacity exercise tolerance. CPET and NIRS are useful tools for detecting early stages of oxidative deficiency in skeletal muscles, disclosing individual impairments in the O_2_ transport and utilization chain.

## Introduction

Amyotrophic lateral sclerosis (ALS) is a fatal neurodegenerative disorder involving alpha motor neurons and abnormal recruitment of motor units, due to lesions in the corticospinal pathways. The resulting clinical manifestations of asthenia, spasticity, and amyotrophy harshly impair functional independence of patients and their quality of life. ALS patients (pALS) have a heterogeneous onset, with increasing fatigability that may begin with impaired activation of limbs, or with dysphagia or dysarthria when the bulbar district is affected first, and with a final failure of respiratory muscles. The appearance of ALS, from the earliest phases of the disease, also typically consists of reduced exercise tolerance until there is complete restriction of activities of daily living^1–3^. The characteristic heterogeneity in exercise tolerance of pALS is related to both the pathologic pattern of motor unit recruitment, and muscle impairment due to disuse of potentially healthy muscles^3, 4^.

The reduced exercise tolerance in pALS (*i*.*e*., the capacity to maintain workloads ranging from habitual activities to rehabilitation exercises) has been associated with mitochondrial dysfunction, both as a direct pathogenic mechanism and as a factor contributing to the exercise limitation^5, 6^. Furthermore, the degree of exercise intolerance in pALS might correlate with the reduction in the number and effectiveness of functional mitochondria able to guarantee an adequate O_2_ extraction at the skeletal muscles^5^. During the early stages (less than 9 months from disease onset), pALS show no evidence of mitochondrial dysfunction. However, this is clearly present with increasing severity and when the disease is finally identified by clinical disability scales^7^. Nevertheless, a subclinical mitochondrial dysfunction may be present in the early stages of ALS and undetectable in resting conditions, only to become apparent with additional external and environmental stress such as exercise^8^. In pALS this phenomenon has not been fully characterized in term of the underlying governing dynamics of oxidative metabolism, and specifically by assessing skeletal muscle oxidative function during exercise. By evaluating the balance between O_2_ delivery and O_2_ extraction at the skeletal muscle level (fulfilling the principle of mass conservation as settled by Fick equation), it is possible to obtain an indirect measure of mitochondrial function. Near infrared spectroscopy (NIRS) is a non-invasive method that can be used to provide an estimation of skeletal muscle fractional O_2_ extraction during exercise in healthy participants or patients^9–12^. According to Ryan and colleagues^13^, NIRS is a useful technique in order to verify oxidative metabolism impairment at the mitochondrial level in the skeletal muscles of pALS. However, although NIRS has been used to assess skeletal muscle oxidative function, the use of low-intensity exercise in this study means that impairments in maximal mitochondrial function may have been missed. Compared to Ryan *et al*. report, we thought to consider an incremental exercise in order to determine the putative impairment of the maximal power of oxidative phosphorylation pathway. Indeed, the expression of integration and efficiency of our body systems is revealed only by exercise, being the resting condition a state where metabolic resources are essentially underused. An efficient chain of transport and utilization of O_2_ from ambient air to mitochondrial level is individually determinable and can be considered our oxidative reserve to face exercise, unless a disease compromises this wealth. In fact, during moderate- or high-intensity physical activities carried out for several minutes, such as activities of daily living in pALS, the oxidative metabolism becomes the prevalent mechanism responsible for adenosine triphosphate (ATP) resynthesis necessary to sustain skeletal muscle functions.

In addition, there is often impairment of the respiratory system in neuromuscular conditions, such as ALS. Nonetheless, even individuals with a well-advanced disease can have a ventilatory reserve sufficient to cope with maximal exercise^6^. In this regard, the respiratory system is able to adapt and triggers mechanisms of spontaneous compensatory respiratory plasticity that preserve breathing capacity, despite an impressive loss of respiratory motor neurons^14^. Once again however, even though pulmonary testing at rest can sometimes identify respiratory muscle impairments, the assessment of respiratory function during exercise is required to understand the contribution of respiratory impairments to the heterogeneity of exercise intolerance in pALS.

The aim of this study was to evaluate, for the first time, the efficiency of the O_2_ transport-utilization chain (*i*.*e*., from lung ventilatory mechanics and diffusion, through cardiovascular O_2_ delivery to tissues, to O_2_ extraction at the muscles) during exhaustive exercise, requiring a large increase in the metabolic demands of skeletal muscles in pALS. The results will contribute to a better understanding of the complex, multifactorial pathogenesis of ALS, and to clarify when there is an early manifestation of oxidative metabolism impairment in pALS. We also investigated if there was a correlation between clinical predictors of ALS progression and the measures obtained by this study, in order to evaluate if determinants of reduced exercise tolerance might match ALS clinical heterogeneity.

## Materials and Methods

### Participants

Following ethical committee approval (protocol #129, 10-JUN-2014, University of Milano-Bicocca) and informed consent, 17 patients diagnosed with “possible” or “probable” ALS according to the revised El Escorial criteria were recruited^15^. They performed an exercise tolerance test at baseline (T0) and 4 months later (T1), and these results were compared with those of 13 healthy age- and sex- matched sedentary controls (CTRL, age 52.1 ± 10.6 yrs.; 11 M/2 F). Furthermore, all procedures and methods were performed in accordance with the relevant international guidelines on exercise testing to reduce any psychological or physical discomfort of participants to a minimum. The data presented in this manuscript are part of the “ME_E_SLA” project (ClinicalTrials.gov Identifier: NCT02548663).

Table 1 shows the demographic and clinical characteristics of pALS at T0. Patients were clinically characterized with the ALS functional rating scale-revised version (ALSFRS-R)^15^. The disease progression index (DPI) was estimated as the loss in ALSFRS-R score over time; i.e., difference of ALSFRS-R scores (T1-T0)/3 months^16^. Nutritional status was evaluated by the Mini Nutritional Assessment (MNA)^17^, where malnutrition ranges from 0 to 16.5, risk of malnutrition from 17 and 23.5, and a normal nutrition status is defined by scores >24 (up to 30). The fat free mass (FFM) was calculated by measuring skinfolds at 7 sites (C10 Plicometer Tanner – Whitehouse; Holtain, Ltd., Crymych, UK), and applying the Jackson Pollock body density equation^18, 19^. The exclusion criteria for this study were: inability to pedal on a cycle-ergometer, unstable cardiac disease, acute infective disorders preventing the execution of the test, or resting arterial blood O_2_ saturation (SaO_2_) < 92%. At T1, 2 pALS dropped out due to apprehension in performing the cardiopulmonary exercise test (CPET, described below), and 1 pALS dropped out due to the caregiver’s personal reasons.

**Table 1:** Demographic and clinical characteristics of patients with amyotrophic lateral sclerosis (pALS).

pALS n = 17			
Age, y	52.2 ± 9.7 (39–70)	Sex, F/M	3/14
BMI, kg/m^2^	25.6 ± 2.9 (18.7–29.9)	EDC, definite/probable	10/7
FFM, kg	60.7 ± 11.2 (36.1–72.5)	Onset, B/S	4/13
ALSFRS-R	37.1 ± 6.2 (26–46)	NIV, no/yes	12/5
DET, months	13.7 ± 11.6 (1–48)	EN, no/yes	0/17
*Disease duration, months	16.7 ± 14.8 (1–48)	Riluzole, no/yes	4/13
DPI	1.2 ± 0.9 (0–3.6)	MNA	20.7 ± 3.7 (11–26)

B = bulbar or S = spinal onset; BMI = body mass index; DET = diagnosis elapsed time (from disease onset); DPI = disease progression index; EDC = El Escorial diagnosis category; EN = enteral nutrition; FFM = fat free mass; ALSFRS-R = ALS Functional Rating Scale-Revised; MNA = mini nutritional assessment index; NIV = non-invasive ventilation. *From the diagnosis to study start. Data are shown as mean ± SD (range).

### Cardiopulmonary exercise test

Each participant underwent to CPET on a cycle-ergometer (Ergomedic Monark LC6: Monark, Varberg-Sweden) under medical supervision, at baseline and only for pASL also after 4 months. Electrocardiography was used in order to detect heart rhythm and frequency by 12-lead electrocardiography monitoring (Quark C12x: Cosmed, Roma-Italy), and SaO_2_ was continuously recorded via pulse oximetry at the finger (RAD 9 Signal Extraction Pulse Oximeter: Masino Corporation, Irvine – California, USA). Confirmation that participants were in a resting condition before all tests was achieved by measuring heart rate (HR), tidal volume (Vt) and respiratory frequency (Rf) (*i*.*e*., pulmonary ventilation, $\dot{{\rm{V}}}$E, in BTPS). After 2 minutes of unloading pedaling the ramp steepness (either 3 or 5 or 10 or 15 W*min^−1^) was chosen empirically according to habitual activities of the pALS, as ascertained by a pre-test interview, and participants cycled until voluntary exhaustion was reached. “Exhaustion” was defined by the inability to maintain cycle rate despite vigorous encouragement by the operators. The Borg scale scored the subjective perceived rate of exertion at exhaustion^20^. Breath-by-breath $\dot{{\rm{V}}}$E, O_2_ uptake ($\dot{{\rm{V}}}$O_2_) and CO_2_ output ($\dot{{\rm{V}}}$CO_2_) were measured by a metabolic cart (Vmax Spectra 229: Sensormedics, Yorba Linda, CA-USA). The peak O_2_ consumption ($\dot{{\rm{V}}}$O_2peak_) was determined: this variable was intended as a descriptor of the overall efficiency of the O_2_ transport and utilization chain, and was used as a measure of exercise tolerance^1^. Capillary blood samples were obtained by puncturing the ear lobe before and at the end of the CPET (during the first 9 minutes of recovery), and blood lactate concentration was measured by a dual-channel analyzer (Biosen C-line Clinic: EKF Diagnostic, Cardiff-England). The peak value measured during 9 minutes of recovery was considered the peak lactate concentration.

### Respiratory tests

Before the CPET each patient was asked to perform a spirometric test in both orthostatism and clinostatism for the determination of forced vital capacity (FVC) and forced expiratory volume in the first second (FEV1). The diffusion lung capacity through the air blood barrier (DLCO) was also measured (Vmax Spectra 229: Sensormedics, Yorba Linda, CA-USA). DLCO measurements were performed at total lung capacity using a single-breath method and having participants inspire a gas mixture containing 0.3% CH_4_, 0.3% CO and 20% O_2_. A visual evaluation of the expected spirometry and DLCO curves was performed, and the average of three consecutive and repeatable measures was used. All variables were expressed with respect to predicted values according to age, sex, and BMI.

### Vastus lateralis near infrared spectroscopy

NIRS is a non-invasive method that allows for the monitoring of tissue oxygenation using the principle that the near-infrared light absorption characteristics of haemoglobin (Hb) and myoglobin depend on their O_2_ saturation at different wavelengths (780 and 850 nm, respectively)^11, 12^. Light in the near-infrared spectrum readily penetrates skin, fat, and bone and is differentially absorbed by heme-containing molecules in underlying tissues - predominantly oxy (Δ[HbO_2_]) and de-oxyhemoglobin (Δ[HHb]). Decreases in Δ[O_2_Hb] and increases in Δ[HHb] were interpreted as evidence of a balance between delivery and extraction relative to the tissues under investigation. A 2-channel continuous wave NIRS was used to detect muscle oxygenation (Nimo: Nirox, Brescia, Italy). This system is not equipped to measure blood flow. The skeletal muscle NIRS probe was positioned on the distal end of the dominant thigh to monitor oxygenation changes in the vastus lateralis muscle. The probe was firmly placed on the skin (~10 cm above the proximal border of patella and 3 cm lateral to the midline of the thigh) and secured with a Velcro strap. The detector–illuminator distance was set at 3.5 cm. Elastic bandages were put around the probe to prevent contamination from localised light. Pen-marks were made over the skin to indicate the margins of the plastic spacer in order to check for any downward sliding of the probe during cycling. Once secured in place, no sliding of the probe was detected for any of the tests. Skinfold thickness at the site of application of the muscle NIRS probe was determined before the exercise protocol and the measured average values of skin and subcutaneous thigh tissue thickness in pALS and CTRL were 16.1 ± 9.2 mm (range 7.1–40.0) and 15.3 ± 8.7 mm (range 4.4–27.4), respectively. At the end of exercise a “physiological calibration” was performed by inducing a transient ischemia of the investigated limb, obtained by applying a wider cuff (tourniquet) to the proximal part of the thigh and inflating to 250–350 mmHg (depending on the participant’s body mass index). This manoeuvre needs 4 to 6 min and can be uncomfortable for some participants. The maximal de-oxygenation was considered when the increase in Δ[HHb] and the decrease in Δ[O_2_Hb] reached a “plateau”. Ischemia was obtained while individuals were sitting on the examination table. Individual extraction capacity was expressed as Δ[HHb]/Δ[HHb]isch.

#### *Study design and statistical methods*

This is an analytic observational case-control study (pALS versus CTRL). Values were expressed as means ± (SD). In order to estimate the necessary sample size, the results from a previous study regarding oxidative impairment in myopathies were used^21^, and a difference of 20% of Δ[HHb]_peak_ in pALS compared to CTRL was a priori hypothesized: we estimated that a sample size of 15 participants would have been sufficient to obtain 80% power (α = 0.05).

A D’Agostino and Pearson omnibus normality test was used to check if values came from a Gaussian distribution. The statistical significance of the difference in mean values between groups was evaluated by unpaired two-tailed Student’s *t*-tests if the data were normally distributed. Otherwise, the Mann-Whitney test was used (95% of confidence level). Regression and correlation analyses were performed using the least squared residuals method. The level of significance was set at p < 0.05. All statistical analyses were performed using a commercially available software package (Prism 6.0: GraphPad, La Jolla, CA, USA).

## Results

### Exercise tolerance and muscle O_2_ extraction

**Figure 1:**
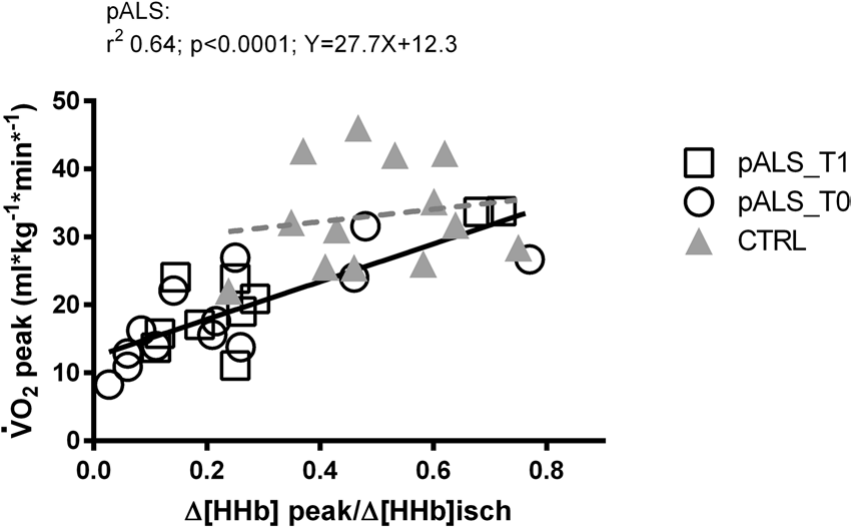
Correlation between exercise tolerance ($\dot{{\rm{V}}}$O_2peak_) and vastus lateralis O_2_ extraction at peak of exercise (Δ[HHb]/Δ[HHb]isch), in pALS (black line) and CTRL (broken line, not significant). White circles: pALS at baseline (T0); white squares: pALS after 4 months (T1); grey triangles: matched healthy CTRL.

At T0, exercise tolerance was significantly reduced ($\dot{{\rm{V}}}$O_2peak_ ~44% lower) in pALS when compared with CTRL (18.5 ± 6.4 *vs* 33.1 ± 7.8 ml*kg^−1^*min^−1^; p < 0.0001, coefficient of variation 35 *vs*. 23%). This was paralleled by a 43% lower peak skeletal muscle oxidative function in pALS vs. CTRL (0.28 ± 0.24 *vs*. 0.49 ± 0.14; p < 0.01, coefficient of variation 86% *vs*. 28%). A linear regression between these variables was found at T0 and T1. Since the slopes and intercepts were not different at T0 and T1, it was possible to use pooled slope and intercept, as shown in Fig. 1 (r^2^ = 0.64, p < 0.0001). This correlation was not present in healthy controls (r^2^ = 0.02, p = 0.59). In Fig. 1 data are shown with the exclusion of 3 participants, due to objective and subjective limits of the NIRS technique^12^: 2 pALS had an excessively thick layer of subcutaneous fat (>1.5 cm) to permit the accurate assessment of skeletal muscle oxidative function; 1 pALS did not tolerate the leg-cuff ischemia maneuver. Two more pALS (1 at T0 and 1 at T1) experienced a technical problem with NIRS during the CPET. Globally, the following variables were significantly reduced at exercise exhaustion in pALS with respect to CTRL: (1) $\dot{{\rm{V}}}$O_2peak_, expressed as percentage of the predicted value according to age, sex, body mass and height, was ~41% lower (64.5% ± 18.4 *vs*. 108.4% ± 24.1, p < 0.0001); (2) $\dot{{\rm{V}}}$E _peak_ was ~46% lower (53.0 ± 25.4 *vs*. 98.0 ± 27.4 l*min^−1^, p < 0.0001); (3) Vt_peak_ was ~36% lower (1.66 ± 0.72 *vs*. 2.62 ± 0.46 l; p < 0.0005); (4) peak power was ~38% lower (87.0 ± 47.0 *vs*. 140.2 ± 64.2 W, p < 0.02); (5) blood lactate concentration was ~45% lower (3.8 ± 1.9 *vs*. 7.0 ± 2.9 mM, p < 0.003).

### Muscle power and lactate concentration

**Figure 2:**
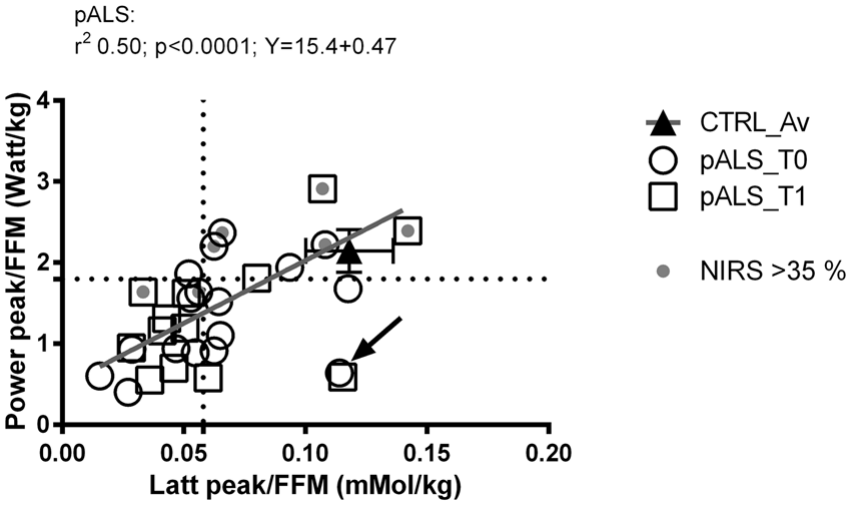
Correlation between peak blood lactate concentration [La] and peak power at exhaustion, normalized for metabolic mass (fatty free mass, FFM), in pALS and CTRL. Four quadrants were defined by drawing dotted lines, according to one SD below the average for CTRL for both variables. White circles: pALS at baseline (T0); white squares: pALS after 4 months (T1); grey triangles: matched healthy CTRL; grey dot: pALS with vastus lateralis O_2_ extraction >35%. Black arrow indicates 1 patient with atypical behavior.

Figure 2 shows the relationship between the capacity to express power during exercise on the cycle ergometer and blood lactate concentration; both variables are expressed relative to the metabolically active body mass (*i*.*e*., FFM, fat free mass). Most of pALS showed values more than one SD below the average for CTRL (left inferior quadrant), pointing to a clear-cut distribution between these two groups of participants. A strong linear regression between power and lactate was present in pALS (r^2^ = 0.50, p < 0.0001): the arrow indicates one single pALS with an atypical blood lactate concentration and a very low peak power output during the exercise. pALS with an efficient skeletal muscle oxidative function, as characterized by peak O_2_ extraction as measured with NIRS (>35), had a peak power output and blood lactate concentration similar to CTRL (Fig. 2).

### Correlations with ALS clinical scores

**Figure 3:**
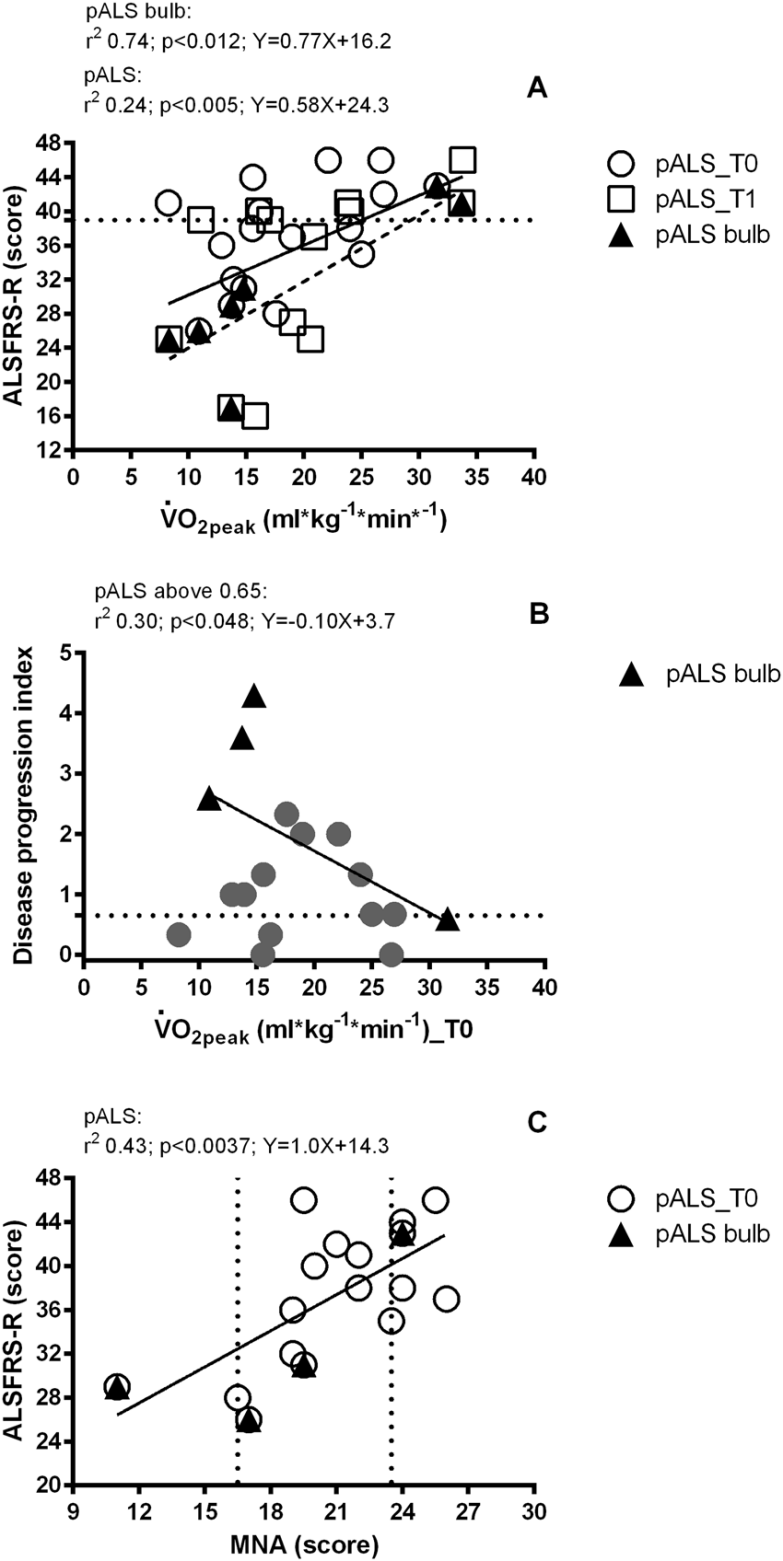
Panel (A) Correlation between $\dot{{\rm{V}}}$O_2_ values at exhaustion and clinical scores of ALS disease (ALSFRS-R questionnaire), recorded at baseline (T0) and after 4 months (T1). Patients with ALSFRS-R score lower than 39 (dotted line) had shorter survival time. The broken line is related only to pALS bulb. Panel (B) Correlation between $\dot{{\rm{V}}}$O_2_ peak and the change in ALSFRS_R score after 4 months (T1–T0). Patients with ALSFRS-R score higher than 0.65 (dotted line) had shorter survival time. Panel (C) Correlation between Mini Nutritional Assessment (MNA) questionnaire scores and ALSFRS-R ﻿at T0. Patients with 16.5 < MNA < 25 had a risk of malnutrition, while patients with MNA < 16.5 were malnourished. White circles: pALS at T0; white squares: pALS at T1; black triangles: pASL with bulbar onset (pALS bulb).

Figure 3A shows a positive linear correlation between ALSFRS-R scores and $\dot{{\rm{V}}}$O_2peak_ (since at T0 and T1 slopes and intercepts were not different, it was possible to calculate a pooled slope and intercept, r^2^ = 0.24, p < 0.005). The linear regression was stronger when only the pALS with bulbar onset were considered (r^2^ = 0,74, p < 0.012). A negative linear correlation was found also between DPI scores above 0.65 (severe progression and shorter survival time) and $\dot{{\rm{V}}}$O_2peak_ (r^2^ = 0.30, p < 0.05; Fig. 3B). A correlation was also observed between MNA score at T0 and ALSFRS-R scores (Fig. 3C). Only 4 pALS (23.5%) had normal MNA scores, while 11 (65%) were at risk of malnutrition and 2 (11.5%) were malnourished.

### Pattern of breathing and respiratory tests

**Figure 4:**
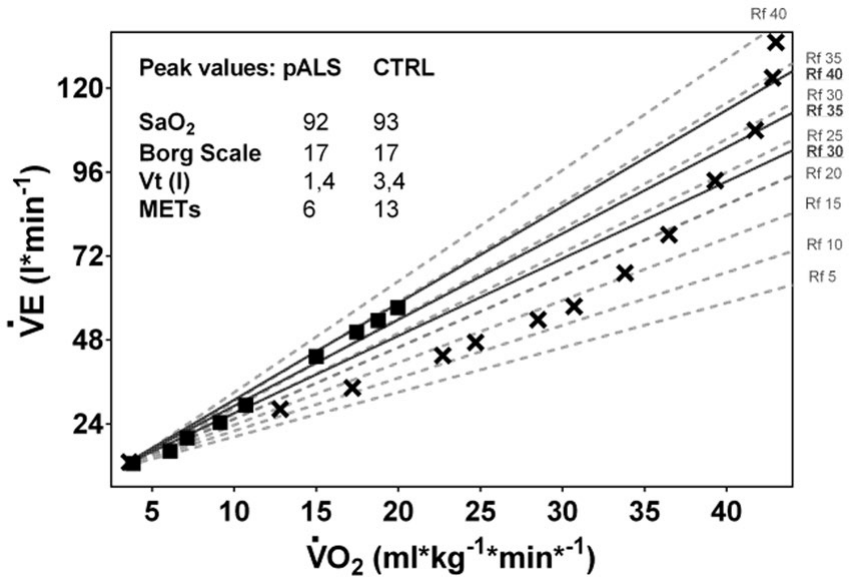
Relationships between $\dot{{\rm{V}}}$O_2_ and ventilation ($\dot{{\rm{V}}}$E) values during exercise in representative participants from the pALS (black squares) and CTRL (crosses) groups. Individually, iso-respiratory frequency (Rf) lines are indicated (pALS, continuous lines; CTRL, broken lines).

pALS presented different pattern of breathing during exercise with respect to CTRL: Figure 4 shows the correlation between $\dot{{\rm{V}}}$O_2_ and pulmonary $\dot{{\rm{V}}}$E during exercise for 2 representative participants (1 pALS and 1 CTRL) that displayed a typical pattern of breathing (*i*.*e*., similar to all the other participants belonging to the same category). The Rf individual iso-lines for both individuals are also shown. Three features can be highlighted on comparing pALS with CTRL: 1) in pALS, $\dot{{\rm{V}}}$E is increased by maintaining, from the onset of exercise up to $\dot{{\rm{V}}}$O_2peak_, a relatively constant and extremely high Rf value (35–40 breaths per min). Conversely, in CTRL Rf started from normal values (10 breath per minutes) and increased constantly until the 75% of $\dot{{\rm{V}}}$O_2peak_ was reached, attaining 40 breath per minute only at the very end of exercise; 2) a higher $\dot{{\rm{V}}}$E was required in pALS, compared to CTRL, to maintain the same $\dot{{\rm{V}}}$O_2_; 3) both $\dot{{\rm{V}}}$O_2peak_ and $\dot{{\rm{V}}}$E_peak_ were clearly limited in pALS compared to CTRL, with the Vt in pALS almost half that of CTRL. The subjective perceived exertion on the Borg scale, and SaO_2_, were the same for pALS and CTRL.

**Figure 5:**
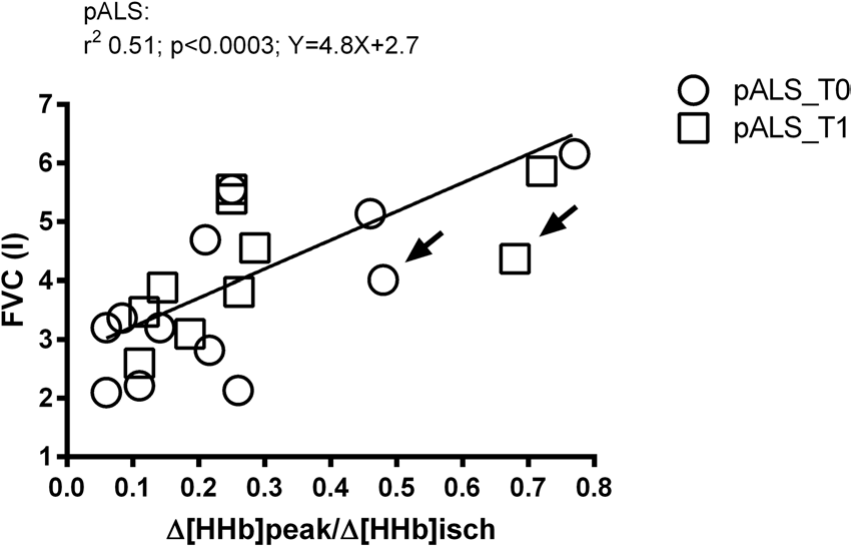
Correlation between orthostatic lung vital capacity (FVC) and vastus lateralis O_2_ extraction (Δ[HHb]/Δ[HHb]isch) at exhaustion, at baseline (T0) and after 4 months (T1), in pALS. White circles: pALS at T0; white squares: pALS at T1. Black arrow indicates 1 pALS with bulbar onset who was incapable of performing fully and correctly the spirometry maneuver.

Figure 5 shows a strong correlation between FVC and muscle oxygen extraction capacity (since at T0 and T1 slopes and intercepts were not different, it was possible to calculate a pooled slope and intercept, r^2^ = 0.51, p < 0.0003): arrows indicate one pALS with bulbar onset incapable of correctly performing the spirometry maneuver because of laryngeal dysfunction.

In Table 2, FVC and FEV1 at T0 and T1, measured in ortho- and clinostatism (expressed as % of predicted value according to sex, age and height) are shown: values ranged from normal to a severe respiratory impairment, with on average, a ~15% deficiency of the same values measured in clinostatism (p < 0.0001 for both parameters); DLCO indices are ~15% reduced if compared to normal values, but not the Khrog index (DLCO relative to the available ventilatory area); impaired FVC and FEV1 (<85% of predicted), in ortho-, but especially in clinostatism, strongly related to the reduced $\dot{{\rm{V}}}$O_2peak_; FVC and FEV1, in ortho-, but especially in clinostatism, were strongly related to the ALSFRS-R scores.

**Table 2:** Relationship between respiratory test parameters obtained during the CPET and clinical scores in pALS.

	pALS_T0	pALS_T1	Regression with $\dot{{\rm{V}}}$O_2_*	only values < 81%^§^	Regression with ALSFRS-R*	only values < 81%^§^
FVC%-ortho	98.1 ± 26.2 (64–137)	98.6 ± 19.3 (69–133)	r^2^ = 0.02 p = 0.42	**r** ^**2**^ ** = 0.44 p = 0.048**	**r** ^**2**^ ** = 0.31 p = 0.001**	r^2^ = 0.01 p = 0.69
FEV1%-ortho	99.9 ± 27.9 (65–144)	104.3 ± 20.1 (75–138)	r^2^ = 0.01 p = 0.51	**r** ^**2**^ ** = 0.47 p = 0.027**	**r** ^**2**^ ** = 0.38 p = 0.0005**	r^2^ = 0.16 p = 0.23
FVC%-clino	84.9 ± 32.1 (24–125)	85.7 ± 31.3 (35–132)	r^2^ = 0.03 p = 0.39	**r** ^**2**^ ** = 0.64 p = 0.001**	**r** ^**2**^ ** = 0.50 p < 0.0001**	**r** ^**2**^ ** = 0.31 p = 0.046**
FEV1%-clino	88.0 ± 32.3 (24–129)	89.5 ± 31.9 (37–124)	r^2^ = 0.02 p = 0.48	**r** ^**2**^ ** = 0.49 p = 0.01**	**r** ^**2**^ ** = 0.54 p < 0.0001**	**r** ^**2**^ ** = 0.27 p = 0.049**
DLCO%	78.5 ± 9.3 (60–89)	86.0 ± 8.4 (73–101)	r^2^ = 0.05 p = 0.33	r^2^ = 0.51 p = 0.58	r^2^ = 0.05 p = 0.34	r^2^ = 0.01 p = 0.11
DLCO/VA%	101.8 ± 14.7 (66–119)	101.2 ± 12.9 (87–117)	r^2^ = 0.02 p = 0.58	N/A	r^2^ = 0.00 p = 0.83	N/A

T0, starting evaluation; T1, second evaluation after 4 months. ^*^Since the slopes and intercepts where not significantly different at T0 and T1, it was possible to calculate one single pooled slope and intercept. ^§^Linear regression analysis was performed again considering only spirometric or DLCO values < 81%. Statistically significant values are reported in bold. N/A, not applicable. FVC, Forced Vital Capacity; FEV1, Forced Expiratory Volume in 1 second, DLCO, Diffusion Lung Capacity.

## Discussion

The novelty of this study resides in having explored the efficiency of the O_2_ delivery and utilization chain in pALS, from the ventilatory phase to muscles extraction when facing a large increase in the metabolic demand during exhaustive exercise. Previous studies provided limited evidence about the skeletal muscle oxidative function of pALS, due to the small increase in metabolic demand (workloads obtained by electrical stimulation)^13^. Our key novel finding consisted of providing an integrated analysis demonstrating impairments of the O_2_ chain in pALS: from reduced pulmonary ventilation, to lower muscle O_2_ oxidative function and VO_2peak_, lactate production and therefore total power output. These data appear relevant to clinical practice, since they provide evidence that the ALSFRS-R score fails to really correlate with the actual metabolic capacity of the patient.

Consistent with the results of Mezzani *et al*.^1^, we report pALS to have a large (~40%) reduction in $\dot{{\rm{V}}}$O_2peak_ once the diagnosis is established. We extend these findings with the observation that $\dot{{\rm{V}}}$O_2peak_ values were 3-fold more heterogeneous in pALS compared to CTRL, presumably due to the variability in the clinical expression of muscle O_2_ extraction, and evolution of this disease.

According to Ryan *et al*., NIRS can be performed successfully in pALS and is a particularly sensitive technique to measure muscle O_2_ function in patients with denervated muscle^13^. In our study, lower NIRS values at exhaustion in pALS were associated with poorer exercise tolerance, and those pALS expressing values within the normal range of oxidative function showed an exercise tolerance that was comparable to CTRL. Nevertheless, even pALS with a similar level of O_2_ extraction compared to CTRL were unable to achieve 100% of their predicted $\dot{{\rm{V}}}$O_2peak_. The strong limitation in exercise tolerance due to a low value of muscle oxidative function is not present in CTRL, suggesting the balance between O_2_ delivery and extraction is preserved.

NIRS measurements could provide an indirect assessment of mithocondrial disfunction in pALS^13^. Our results suggest that in pALS, a reduced O_2_ extraction at the skeletal muscles could be related to impaired mitochondrial oxidative function, possibly in a manner that is independent from motor neuron impairment^22^. As also reported by Al Sarray and colleagues^22^. our results support the view that additional stress (*e*.*g*., inflammation or, in our study, exercise) could unmask sub-clinical mitochondrial dysfunction in the muscle of pALS. Accordingly, exercise could provide an additional clinical tool to understand the complex pathogenesis of ALS.

In this study, both lactate concentration and power output normalized to the FFM was partially preserved in pALS with the highest values of muscle O_2_ extraction (as assessed by NIRS). As already observed in mitochondrial myopathies^23^, the lower blood lactate accumulation/concentration for the same workload and metabolic active mass (FFM) in pALS can be considered an index of an enhanced oxidative phosphorylation. Yet, in our pALS group, the lower lactate concentrations were found in patients displaying lower ability to extract O_2_ from their muscle. In pALS, these results suggest an individual heterogeneity in the expression of motor neuron disease (non-oxidative/oxidative motor unit recruitment), and a different contribution of less/more efficient oxidative fibers (Fig. 2). This is consistent with the observation that motor neuron death in pALS is different across motor pools^24^. The mechanisms underlying this heterogeneous response remain unknown, but it would be important to identify possible cellular/molecular differences, including diversities in oxidative metabolism, between susceptible versus resistant motor unit pools. NIRS evaluation in pALS could effectively explore oxidative metabolism and characterize the limits of oxidative function affecting individual exercise tolerance: it will be of interest to evaluate in a larger group of patients if a repeated follow up of the $\dot{{\rm{V}}}$O_2peak_ versus O_2_ extraction relationship could have a prognostic value in pALS.

The CPET, in association with NIRS measurements, seems to add new information to assist the clinical follow up in pALS by examining the skeletal muscle oxidative function. The coefficient of variation of $\dot{{\rm{V}}}$O_2peak_ values was especially elevated in patients with ALSFRS-R scores above 39, suggesting that in pALS, the clinical evaluation alone can hardly discriminate the real exercise tolerance otherwise measurable by CPET: some pALS considered “inefficient” at the ALSFRS-R showed a still residual capacity from the $\dot{{\rm{V}}}$O_2peak_ point of view. Furthermore, only DPI above 0.65 are, in point of fact, related to a poor exercise tolerance, but some pALS with a low progression of disease (<0.25) were severely impaired regarding exercise tolerance. Kollewe and colleagues showed that 44% of pALS with a DPI < 0.25 will survive at least 5 years: our findings might possibly give reasons about those patients that, having the same DPI, nevertheless escape from this favorable survival rate^16^. In fact, the ALSFRS-R score alone, if compared to $\dot{{\rm{V}}}$O_2peak_ measure, seems to be less sensitive to the real impairment of the efficiency of the O_2_ transport and utilization chain and is –therefore– not useful to practice any hypothetical tailored exercise trial, aimed at individual and sustainable workloads. An exception to this consideration might be found for those pALS with bulbar onset where the ALSFRS-R scores were strongly correlated to $\dot{{\rm{V}}}$O_2peak_ values. Notably, about 80% of pALS in this study had a risk of malnutrition or were malnourished, as revealed by the MNA evaluation. Both MNA and plicometry could be useful to define a correct dietary intake from the earliest onset of clinical manifestation, in order to counteract the hypermetabolic state characteristic of the disease^25^, and especially if a tailored exercise program is advocated.

We also observed, especially for pALS with a severe impairment in clinostatism, a relationship between FVC and exercise tolerance. As known, FVC is a well-known prognostic factor in pALS, where FVC lower than 81% at first visit had a shorter median survival within 27 months^16^. However, we provide new information that FVC is also an important determinant of the reduced exercise tolerance in pALS due to the relationship between the reduced FVC and the muscle capacity of O_2_ extraction at the peak of exercise.

Since the primary insult in ALS is motor neuron death, the extent of the direct involvement of the respiratory muscle may be heterogeneous and the relatively spared respiratory motor neuron pools may compensate for the failure of the impaired ones^14^. Thus, we hypothesized that in some pALS respiratory muscle impairment may only become apparent when challenged during exercise. We observed a lower $\dot{{\rm{V}}}$E in pALS with the increase in VO_2_, essentially reflecting the inability to increase Vt, not compensated by the increase in Rf. This finding may reflect a reduced efficiency of inspiratory muscles to face the remarkable energy requirement to increase Vt, as well as their recruitment. While a NIRS evaluation directly on respiratory muscles is needed in order to effectively measure their oxidative function, it remains possible that the restrictive tendency of the chest wall compliance due to the weakness of respiratory muscles in pALS^6^, would require an increased metabolic demand by the respiratory muscles. Thus, we may reason that reduced oxidative efficiency of respiratory muscles (as also seen in deconditioning) could be a limit when an adequate ventilatory reserve is necessary to maintain the ventilatory response to exercise.

The present data, revealing an inefficient skeletal muscle oxidative function can be explained on the basis of an incomplete activation of muscle fibers, due to the progressive impairment of motor neuron recruitment. Yet, a muscle deconditioning process related to exercise intolerance can further contribute to the metabolic derangement. We consider this point critical, because specific mechanisms of exercise limitation in pALS are related to the requirement of a reduced pool of motor neurons to innervate a greater number of muscle fibers in the large muscle masses of the limbs and respiratory muscles. These macro-motor units become progressively less efficient due to the drop out of the chronically overloaded lower motor neurons and to the neuromuscular conduction defect along the reinnervated nerve sprouts^6^. Thus, our data support the hypothesis that severity of denervation and time since onset of the disease progressively impairing the oxidative function, both affect exercise tolerance and can be fully revealed only when the intensity of exercise requires a large increase in the metabolic demands of skeletal muscles in pALS.

### Limitations

An intrinsic limitation of NIRS evaluation is the thickness of the fat layer under the probe positioned on the investigated muscle, as seen in sedentary women on the thigh or in participants displaying severe atrophy^12^. In all pALS, only the vastus lateralis muscle with the least functional impairment was investigated in order to avoid the possibility of severe atrophy under the probe. In addition, we placed the probe over the deeper muscle regions in the quadriceps muscle (where the greater proportion of oxidative fibers with more oxidative energy metabolism are presented)^12^. Otherwise, the area of muscle investigated by the NIRS probe may not represent, in terms of fiber types and motor unit recruitment pattern and/or fiber blood perfusion, a reliable assessment of the whole muscle^12, 13^. However, it should be considered that the same limitation is intrinsically associated with all methods that investigate only a small portion of a muscle, including a muscle biopsy.

## Conclusion

In summary, because of the difficulties in early diagnosis, and in the evaluation of disease progression, there is a growing need to identify clinically useful methods that can also aid to assess the impact of tailored rehabilitation interventions in pALS. Our results suggest that ALS is a disease with a multifactorial damage, not only related to the impaired recruitment of motor neurons. A heterogeneous manifestation of inefficient oxidative metabolism, at skeletal muscle level, is deeply related to the individual exercise capacity and function in pALS. Assessment of $\dot{{\rm{V}}}$O_2peak_ and muscle oxygen extraction by NIRS might represent advantageous and non-invasive tools to evaluate the heterogeneous clinical expression of pALS. This findings have a putative prognostic value to be confirmed with a longitudinal evaluation.

### Data availability

All raw data are available upon request to the corresponding Author (FL).
